# A region-based palliative care intervention trial using the mixed-method approach: Japan OPTIM study

**DOI:** 10.1186/1472-684X-11-2

**Published:** 2012-01-11

**Authors:** Tatsuya Morita, Mitsunori Miyashita, Akemi Yamagishi, Nobuya Akizuki, Yoshiyuki Kizawa, Yutaka Shirahige, Miki Akiyama, Kei Hirai, Motohiro Matoba, Masako Yamada, Taketoshi Matsumoto, Takuhiro Yamaguchi, Kenji Eguchi

**Affiliations:** 1 Department of Palliative and Supportive Care, Palliative Care Team, and Seirei Hospice, Seirei Mikatahara, General Hospital 3453 Mikatahara-cho, Hamamatsu, Shizuoka 433-8558, Japan; 2 Department of Adult Nursing/Palliative Care Nursing, School of Health Sciences and Nursing, Graduate, School of Medicine, The University of Tokyo, 7-3-1, Hongo, Bunkyo-ku, Tokyo 113-0033, Japan; 3 Department of Adult Nursing/Palliative Care Nursing, School of Health Sciences and Nursing, Graduate, School of Medicine, The University of Tokyo, 7-3-1, Hongo, Bunkyo-ku, Tokyo 113-0033, Japan; 4 Psycho-Oncology Division, Research Center for Innovative Oncology, National Cancer Center Hospital East 6-5-1, Kashiwanoha, Kashiwa, Chiba 277-8577, Japan; 5 Department of Medical Social Service, Center for Palliative and Supportive Care, Graduate School, University of Tsukuba, 1-1-1 Ten-nohdai, Tsukuba 305-8575, Japan; 6 Shirahige Clinic, 1-13-28, Katafuchi, Nagasaki, Nagasaki 850-0003, Japan; 7 Faculty of Policy Management, Keio University, 5322 Endo, Fujisawa-shi, Kanagawa 252-8520, Japan; 8 Graduate School of Human Sciences; Center for the Study of Communication-Design; Department of Complementary and Alternative Medicine, Graduate School of Medicine, Osaka University, 1-2, Yamadaoka, Suita, Osaka 565-0871, Japan; 9 Department of Palliative Medicine and Psycho-Oncology, National Cancer Center, 5-1-1 Tsukiji, Chuo-ku, Tokyo 104-0045, Japan; 10 Research Center for Development of Nursing Practice, St. Luke's College of Nursing 3-8-5 Tsukiji, Chuo-ku, Tokyo 104-0045, Japan; 11 Division of Respiratory Disease, Head of Regional Support, Kumamoto Takumadai Hospital, 1-14-27 Onoue, Kumamoto-city, Kumamoto 862-0913, Japan; 12 Department of Clinical Trial Data Management, Graduate School of Medicine, The University of Tokyo, Hospital, 7-3-1, Hongo, Bunkyo-ku, Tokyo 113-0033, Japan; 13 School of Medicine, Tokai University, 143 Shimokasuya, Isehara, Kanagawa 259-1143, Japan

## Abstract

**Background:**

Disseminating palliative care is a critical task throughout the world. Several outcome studies explored the effects of regional palliative care programs on a variety of end-points, and some qualitative studies investigated the process of developing community palliative care networks. These studies provide important insights into the potential benefits of regional palliative care programs, but the clinical implications are still limited, because: 1) many interventions included fundamental changes in the structure of the health care system, and, thus, the results would not be applicable for many regions where structural changes are difficult or unfeasible; 2) patient-oriented outcomes were not measured or explored only in a small number of populations, and interpretation of the results from a patient's view is difficult; and 3) no studies adopted a mixed-method approach using both quantitative and qualitative methodologies to interpret the complex phenomenon from multidimensional perspectives.

**Methods/designs:**

This is a mixed-method regional intervention trial, consisting of a pre-post outcome study and qualitative process studies. The primary aim of the pre-post outcome study is to evaluate the change in the number of home deaths, use of specialized palliative care services, patient-reported quality of palliative care, and family-reported quality of palliative care after regional palliative care intervention. The secondary aim is to explore the changes in a variety of outcomes, including patients' quality of life, pain intensity, family care burden, and physicians' and nurses' knowledge, difficulties, and self-perceived practice. Outcome measurements used in this study include the Care Evaluation Scale, Good Death Inventory, Brief pain Inventory, Caregiving Consequence Inventory, Sense of Security Scale, Palliative Care Knowledge test, Palliative Care Difficulties Scale, and Palliative Care Self-reported Practice Scale. Study populations are a nearly representative sample of advanced cancer patients, bereaved family members, physicians, and nurses in the region.

Qualitative process studies consist of 3 studies with each aim: 1) to describe the process in developing regional palliative care in each local context, 2) to understand how and why the regional palliative care program led to changes in the region and to propose a model for shaping regional palliative care, and 3) to systemically collect the barriers of palliative care at a regional level and potential resolutions. The study methodology is a case descriptive study, a grounded theory approach based on interviews, and a content analysis based on systemically collected data, respectively.

**Discussion:**

This study is, to our knowledge, one of the most comprehensive evaluations of a region-based palliative care intervention program. This study has 3 unique aspects: 1) it measures a wide range of outcomes, including quality of care and quality of life measures specifically designed for palliative care populations, whether patients died where they actually preferred, the changes in physicians and nurses at a regional level; 2) adopts qualitative studies along with quantitative evaluations; and 3) the intervention is without a fundamental change in health care systems. A comprehensive understanding of the findings in this study will contribute to a deeper insight into how to develop community palliative care.

**Trial Registration:**

UMIN Clinical Trials Registry (UMIN-CTR), Japan, UMIN000001274.

## Background

Palliative care is an essential part of integrated cancer treatment [1]. Palliative care should be provided throughout an entire region, and several outcome studies have explored the effects of regional palliative care programs on places of death, the use of palliative care resources, patient-reported outcomes, family-reported outcomes, and cost [2-9]. In a cluster randomized controlled trial, a regional palliative care intervention, including developing a specialized inpatient palliative care service at an academic hospital, strengthening the cooperation between specialized palliative care and community health care services, developing clinical guidelines, and educational programs for community health care providers, contributed to an increase in the number of home deaths and higher levels of family satisfaction, but the patient-reported quality of life, measured by the EORTC-C30, was not significantly different [2-4]. A pioneer work as a regional palliative care model, the Edmonton program, observed that constructing a novel regional system, including a regional palliative care office to coordinate palliative care activities at specialist and community levels throughout the region, resulted in an increased number of home deaths and use of specialized palliative care services [5,6]. A recent palliative care quality improvement project in Ontario included developing and disseminating standard clinical tools for collaborative care planning and symptom assessment [7,8]. The audit study from this project demonstrated an increased documentation of symptoms and decreased use of emergency visits, while the symptom intensity and family satisfaction did not significantly improve. In addition, the Catalonia WHO demonstration project demonstrated an increase in the quantity and variety of specialized palliative care services and potential cost-saving effects [9]. More recently, the U.K. implemented the Gold Standards Framework, stressing communication and coordination in the community through developing a palliative care patient registry and regular meetings [10-13]. Qualitative studies suggest the most important benefit of the Gold Standards Framework is facilitating communication among health care professionals in the community, although the direct effects on patient and/or family outcomes were formally unmeasured. Multiple studies from Canada, the Netherlands, and Australia which investigated the process of developing community palliative care networks again revealed the perceived importance of an increase in personal and formal contacts in health care professionals [14-16].

These studies provide important insights into the potential benefits of regional palliative care programs, but the clinical implications are still limited, because: 1) the interventions included a fundamental change in the structure of the health care system (Norway, Edmonton, Catalonia, and the U.K.), and, thus, the results would not be applicable for many regions where structural changes are difficult or unfeasible; 2) patient-oriented outcomes were not measured or explored only in a small number of populations, and interpretation of the results from a patient's view is difficult; and 3) no studies adopted the mixed-method approach using both quantitative and qualitative methodologies to interpret the complex phenomenon from multidimensional perspectives [17-20].

We believe, therefore, that a new study should include: 1) an intervention program available for many regions without fundamental changes to the health care system, 2) adequate patient-oriented outcomes, and 3) qualitative studies along with quantitative evaluation.

The aim of this presentation is to describe a study protocol of a region-based palliative care intervention trial using the mixed-method approach: the Outreach Palliative care Trial of Integrated Model (OPTIM study) from Japan.

## Methods and Design

### Overview and aims

This is a regional intervention trial, consisting of a pre-post outcome study and qualitative process studies. Initially, this study was designed as a cluster randomized controlled trial, but we have decided to adopt a mixed-method design because: 1) intervention itself should be applied to all populations over the country and a clear distinction between intervention and control groups is difficult [20], 2) a concealment problem is likely to occur [2-4], and 3) the most important mission at a national level is not to clarify if one specific intervention actually changes outcomes, but to obtain comprehensive insights into how to disseminate palliative care throughout the country [17,18].

The primary aim of the pre-post outcome study is to evaluate the change in the number of home deaths, use of specialized palliative care services, patient-reported quality of palliative care, and family-reported quality of palliative care after the regional palliative care program. The secondary aims are to explore the changes in a variety of outcomes, including the distribution of locations of death; patient- and/or family-reported quality of life, whether patients died where they actually preferred, time spent at home, satisfaction, pain, care burden; knowledge, belief, and concerns about palliative care; and knowledge, difficulties, and self-reported practice about palliative care of physicians and nurses. Qualitative process studies are performed to obtain a deep insight into how and why the regional palliative care program does or does not work.

Data for the pre-post outcome study were collected in 2007-2008 as pre-intervention data and in 2010-2011 as post-intervention data. Data for qualitative studies were collected throughout the study periods.

Ethical and scientific validity was confirmed by the institutional review board of this study and all participating institutions.

### Setting

To explore the potential influence of the variations in the existing health care system, we have decided to conduct this trial in 4 regions with different palliative care systems across Japan: Tsuruoka (170,000 population, Yamagata Prefecture), Kashiwa (670,000 population, Chiba Prefecture), Hamamatsu (820,000 population, Shizuoka Prefecture), and Nagasaki (450,000 population, Nagasaki Prefecture). Kashiwa and Hamamatsu have specialized hospital palliative care teams in a cancer center and general hospitals, respectively; Nagasaki has a coordinated palliative care system for home patients in addition to hospital palliative care teams; and Tsuruoka has no formal specialized palliative care service at the beginning of the study.

### Interventions

#### Designing interventions

Interventions were designed on the basis of literature review, preliminary survey, and discussion among the researchers and clinical practitioners from the study regions [21-33].

To construct a conceptual framework, we initially reviewed the existing domestic and international literature available to identify barriers to provide quality palliative care [21-23], and performed a preliminary survey of 8,000 general public and 8,000 medical health care providers in the 4 regions before planning the interventions [24]. The task force then drafted the intervention protocol with close collaborative with representative health care providers from the 4 regions, and has finalized intervention protocol. The intervention protocol describes the minimum requirements for this study to allow it to meet the specific situations of regions [19,20].

To deliver the intervention, each region is asked to establish a "regional palliative care center" with several local leaders, who receive a start-up 2-day workshop from the study team before intervention with continuous follow-up. Local leaders include physicians, nurses, and medical social workers who have already been working as a clinical specialist in the region. Furthermore, local leaders foster link-staffs at each regional level. To facilitate interventions, meetings among local leaders are planned to be held about 25 times during this study period; a certified community nurse visits each region and followed up by telephone and e-mail as a facilitator; and interactive conferences among link-staffs from the 4 regions are held 3 times.

The interventions include 4 areas: 1) to improve the knowledge and skills of palliative care providers, 2) to increase the availability of specialized palliative care services for community patients, 3) to coordinate community palliative care resources, and 4) to provide appropriate information about palliative care to the general public, patients, and families. We designed all interventions not to require a fundamental change in the health care system, that is, to optimize the resources within the region.

To investigate the actual implementation, we regularly monitor the intensity of interventions by telephone and visiting the intervention area [18]. In addition, in the surveys, we investigate the levels of exposure to each intervention (e.g., whether they used or noticed materials, or they participated in workshops).

#### Specific interventions

To improve the knowledge and skills of palliative care providers, we have prepared a pocket-size manual of palliative care (a book and videos) and 13 assessment tools (12 educational pamphlets for patients and families for each symptom, such as pain; and 1 comprehensive assessment tool). These are provided with printed materials and a web site. The local leaders are asked to disseminate these materials and hold an interactive workshop to educate them on how to use these materials [25-28].

To increase the availability of specialized palliative care services for community patients, each region is asked to establish a community palliative care team through optimizing the existing resources, because, at the time of the study, such community palliative care teams are not available in Japan. In addition, the community palliative care team provides outreach educational visits for community intuitions [29].

To coordinate community palliative care resources, each region is asked to establish a "regional palliative care center". The regional palliative care center is then asked to hold a multidisciplinary conference to develop collaborative relationships among health care workers in the region, and share and resolve problems [10,14-16]. In addition, local leaders facilitate the use of patient-held-records to maintain continuity of care [30], and facilitate the introduction of a discharge planning system for all hospitals in the region [31].

To provide appropriate information about palliative care, we have prepared a hand-sized leaflet, note-sized leaflets, posters, and DVDs about palliative care, and ask local leaders to put them in public and health care institutions [32,33]. In addition, local leaders ask public libraries to provide a "book set" (a set of 100 books about palliative care), and provide workshops for the general public. Target themes identified as barriers include the misconception about cancer pain and opioids, palliative care, and death at home [33].

### Measurements

Questionnaires are sent to patients, bereaved family members, physicians, and nurses recruited consecutively following the inclusion criteria by mail. We intend to obtain the sample as a nearly representative sample of each region as much as possible.

#### Subjects

##### Patients

Due to the lack of an established method to identify all cancer patients living in a specific area in Japan, we identify all hospitals in the study areas with reference to hospital lists from the Japan Hospital Association, the largest authorized organization of hospitals in Japan, and the local resource information. In the pre-intervention survey, we obtained the participation of a total of 23 of 34 hospitals treating cancer patients (8,964 beds of 11,033 beds, 81%).

Inclusion criteria are: 1) adult cancer patients with a primary tumor site in either the lung, esophagus, stomach, colon, rectum, pancreas, liver, biliary system, kidney, prostate, bladder, breast, ovary, or uterus, 2) presence of metastatic or recurrent cancer, 3) outpatient visits to the oncology department or each specialty division, such as respiratory medicine for lung cancer patients (not palliative care division only), and 4) informed of malignancy. We have determined to exclude malignancy of the brain, blood, central nervous system, neck, soft tissue, and other uncommon primary sites, due to the infrequent prevalence and increased technical difficulties in patient recruitment. We have decided to examine only patients who were informed of malignancy, because we use the term "cancer" in the questionnaire. Exclusion criteria include: 1) inability of the patient to complete the questionnaire (dementia, cognitive failure, psychiatric illness, language difficulty, or visual loss), 2) severe emotional distress of the patient as determined by the principal treating physicians, and 3) poor physical condition unable to complete the questionnaire.

##### Bereaved family

Due to the legal limitation of a mortality-follow back survey, we identify all hospitals in the study areas in the same way as the patient survey, and general practice clinics with experience of caring for terminally ill cancer patients with reference to the local resource information.

Inclusion criteria for this bereaved family survey are: 1) bereaved adult family members of an adult cancer patient who died in the institution or at home (one family member was selected for each patient), 2) a primary tumor site of either the lung, esophagus, stomach, colon, rectum, pancreas, liver, biliary system, kidney, prostate, bladder, breast, ovary, or uterus, 3) received medical treatments on at least 3 or more days by the institution, and 4) informed of malignancy. Exclusion criteria include: 1) incapacity to complete the questionnaire (dementia, cognitive failure, psychiatric illness, language difficulty, or visual loss), 2) severe emotional distress of the family as determined by the principal treating physicians, 3) treatment-associated death or death from commodity, 4) death in intensive care units, and 5) family member unavailable.

##### Physicians and nurses

We identify hospitals treating cancer patients in the same way as the patient survey, all general practice clinics, and district nurse services.

Inclusion criteria for the physician and nurse survey are: 1) hospital physicians and nurses working at cancer-related branches for at least 3 years (internal medicine, surgery, respiratory medicine, gastro-enterology, urology, breast cancer, gynecology, hematology, radiation oncology, clinical and medical oncology, otopharyncology, and palliative medicine), 2) representative physicians of general practice clinics, and 3) all district nurses.

#### Outcome Measures (Table 1)

**Table 1:** Outcome measures

	Primary end-points	Secondary end-points
Location of death	Home death	Distribution of location of death (home, hospital, palliative care units, nursing home)

Use of specialized palliative care services Patients	Number of patients who received specialized palliative care services	Backgrounds of patients referred to specialized palliative care services

**Patients**		
Quality of palliative care	Total score of 3 subscales of "physical care provided by physicians", "physical care provided by nurses", "psycho-existential care) of the Care Evaluation Scale	Care Evaluation Scale -physical care provided by physicians -physical care provided by nurses -psycho-existential care -help with decision-making -coordination/consistency of care
Quality of life		Good Death Inventory -physical and psychological comfort -living in a favorite place -maintaining hope and pleasure -having a good relationship with medical staff -not feeling a burden to others -having a good relationship with the family -having independence -having environmental comfort -being respected as an individual -a feeling of fulfillment at life's completion
Pain		Brief Pain Inventory
Satisfaction		Satisfaction scale
Knowledge, beliefs, and concerns		Knowledge of opioids, Beliefs about palliative care, Concerns about home care
Feelings of support and security about cancer care in the region Bereaved family members		Sense of Security Scale

**Bereaved family members**		
Quality of palliative care	Total score of 3 subscales of "physical care provided by physicians", "physical care provided by nurses", "psycho-existential care) of the Care Evaluation Scale	Care Evaluation Scale -same as patient, and, -help with decision making for family -family care
Quality of life of the patient (proxy)		Good Death Inventory (same as patient)
Satisfaction		Satisfaction scale
Knowledge, beliefs, and concerns		Knowledge of opioids, Beliefs about palliative care, Concerns about home care
Feelings of support and security about cancer care in the region		Sense of Security Scale
Care burden		Caregiving Consequence Inventory -Burden
Time spent at home		Time spent at home

**Physicians and nurses**		Palliative Care Knowledge Test -philosophy, pain, dyspnea, delirium, and gastro-intestinal symptoms
		Palliative Care Difficulty Scale -expert support, alleviating symptoms, community coordination, communication in multidisciplinary teams, and communication with patients and families
		Palliative Care Self-reported practice Scale

We have determined that this study adopt 4 primary end-points due to the complex nature of the intervention: 1) number of home deaths, 2) number of patients who received specialized palliative care services, 3) patient-reported quality of palliative care, and 4) bereaved family-reported quality of palliative care [20]. In addition, we adopt multiple end-points to interpret the results from multiple perspectives.

##### Location of death

We record the number of cancer patients who died at home, hospitals, and nursing homes from the national government registry every year, and, further, the number of patients who died in palliative care units from each palliative care unit. As the reference data, we obtain the average rate of home death in Japan during this study period. The rationale of setting the number of home deaths as one of the primary end-points is that, while many patients want to die at home, home deaths actually occur at a rate of only 6.0% in Japan [24].

##### Use of specialized palliative care services

The rationale of setting the number of patients who received specialized palliative care services as one of the primary end-points is that multiple studies revealed the beneficial effects of specialized palliative care services on patient outcomes [34,35], and, thus, we believe it is reasonable to assume that higher involvement in specialized palliative care services would result in the improvement in patient outcomes [36]. To calculate the number of patients who received specialized palliative care services, we initially identify all specialized palliative care services, and ask each service to provide a complete patient list every year. The specialized palliative care service is defined as "specialized palliative care provided by palliative care specialists", including: 1) palliative care unit, 2) hospital palliative care team, 3) community palliative care team, 4) outpatient palliative care clinic, and 4) home palliative care team.

The number of patients who received specialized palliative care services is defined as the total of number of patients listed in each specialized palliative care service, and, thus, if one patient received two types of specialized palliative care service, the number of uses of specialized palliative care services is two. Although we have acknowledged the non-duplicated counting is ideal, we gave up on this calculation because not all participating institutions allow providing patient data beyond the institutions due to privacy issues. The ratio of the number of patients who received specialized palliative care services to all cancer death was calculated.

In addition, to explore whether "early palliative care" is realized, the backgrounds of patients referred to specialized palliative care services, such as the performance status, status of disease-modifying treatment, and consultation aims, are obtained from the patient lists.

##### Quality of palliative care

The quality of palliative care is measured by both patients and bereaved families using the Care Evaluation Scale, a well-validated and the most commonly used measurement tool to quantify the user-perceived quality of palliative care in Japan [35]. The psychometric properties are established in both patients and bereaved family members [37,38]. The full version of the Care Evaluation Scale consists of 8 subscales for patients and 10 subscales for families with a 6-point Likert-type scale from "1: improvement is not necessary at all" to "6: highly necessary". One item example is "doctors dealt promptly with discomforting symptoms of the patient". For this study, we have excluded 3 subscales, environment, cost, and availability, because the intervention does not intend to change these outcomes. For the primary end-points, we use 3 subscales (physical care provided by physicians, physical care provided by nurses, and psycho-existential care) as a single scale, because this directly measures the degree to which patients/family members evaluate medical professionals respond to patients' physical and psychological distress. All subscales of the Care Evaluation Scale are used for the secondary end-points.

##### Quality of life

Quality of life is measured by both patients and bereaved families using the Good Death Inventory, a specific measure of the quality of life of Japanese patients with advanced cancer [39,40]. We have decided to use the Good Death Inventory, not common tools such as EORTC or FACT, because: 1) we intend to investigate broader areas of quality of life Japanese palliative care populations regard as important, especially psycho-existential components [41,42], and 2) existing quality of life measures largely depend on patient functional levels and previous studies failed to detect potentially beneficial effects of intervention [4]. The full version of this scale consists of 10 domains with a 7-point Likert-type scale from "1: strongly disagree" to "7: strongly agree". One item example is "I am free from physical distress". The subscale "living in a favorite place" includes "(the patient) is able to stay at his/her favorite place", and, thus, we can analyze not only the death location but also whether the death location was a preferred place of death of the patients [2].

##### Satisfaction

Satisfaction of the patient and family with medical care is measured using a single item scale: "Are you satisfied with the medical care you currently receive?"with a 6-point Likert-type scale varying from "1: very dissatisfied" to "6: very satisfied".

##### Pain intensity

Pain intensity of the patients is measured using the Japanese version of the Brief Pain Inventory, with a score given for the pain at its worst (0-10), at its best (0-10) and a score for the average pain felt (0-10) over the previous 24 hours. The reliability and validity in Japanese populations has been established [43].

##### Knowledge, perceptions, and concerns about palliative care

Knowledge, perceptions, and concerns about palliative care of the patients and families is measured using 10 items, similar to previous surveys [33], on a 5-point Likert-type scale from 1:strongly disagree to 5: strongly agree. Knowledge of opioids is examined using 2 items: "opioids can relieve most pain caused by cancer" and "opioids are addictive and/or shorten life". Beliefs about palliative care are examined using 3 items: "palliative care relieves pain and distress", "palliative care is provided along with chemotherapy and/or radiation therapy", and "palliative care is only for terminally ill patients". Concerns about homecare are examined based on 5 items: "pain can be alleviated as effectively through home-visit services as it can at the hospital", " home - visit services cannot respond to sudden changes in a patient's condition", "it is hard to find home-visiting physicians", and "being taken care of at home puts a burden on the family".

##### Feelings of support and security regarding cancer care in the region

Feelings of support and security regarding cancer care in the region are measured by patients and families using the newly developed Sense of Security Scale [44]. This is 5-item scale to assess feelings of support and security concerning cancer care in a region. One item example is "I would feel secure as a variety of medical care services are available in the region".

##### Care burden

Care burden is measured using the Caregiving Consequences Inventory [45]. The Caregiving Consequences Inventory is developed to quantify caregiving consequences from a bereaved family member's perspective, and it consists of 4 positive domains and 1 burden domain. For this study, the burden domain is used.

##### Time spent at home

Due to a lack of administrative data available to calculate time spent at home, we ask bereaved family members the time spent at home during the last 1 and 3 months.

##### Knowledge, difficulty, and self-perceived practice of palliative care of physicians and nurses

Physician- and nurse-reported knowledge is measured using Palliative Care Knowledge Test [46]. This scale consists of 5 subscales, with correct, incorrect, and do not know responses.

Physician- and nurse-reported difficulty of palliative care is measured with the Palliative Care Difficulty Scale, a validated tool to quantify the levels of difficulty when health professionals provide palliative care [47]. This scale consists of 5 subscales with a 5-point Likert-type scale from 1: never to 5: very much. One item example is "it is difficult to get support from experts on alleviating symptoms".

Physician- and nurse-reported self-perceived practice is measured employing the Palliative Care Self-reported Practice Scale, a validated tool to quantify the levels of adherence to recommended practices in palliative care fields^47^. This scale consists of 6 subscales with a 5-point Likert-type scale from 1: never to 5: very much. One item example is "I routinely inquire about the family's concerns in the dying phase".

##### Quality indicators

As quality indicators as region-level palliative care, we collect 20 quality indicators from nation-level administrative database, such as opioids consumption, the number of home care service, the number of palliative care specialists, and the number of palliative care units.

### Qualitative studies

Qualitative studies consisting of 3 studies.

#### Descriptive study

A descriptive study is performed to describe the process in developing regional palliative care in each local context. The study methodology is descriptive case studies, and this includes a variety of materials each region has made or arranged for local interventions.

#### Interview study

This study is performed to understand the process of how and why the regional palliative care program makes changes in the region. The ultimate purpose of this study is to propose a model for shaping regional palliative care. The research methodology is a grounded theory approach, and the data source is in-depth interviews with health care professionals.

#### Systematic collection of barriers and potential resolutions

This study is performed to systemically identify the barriers of palliative care at a regional level and potential resolutions. The research methodology is content analyses, and the data source is multiple focus-groups repeatedly conducted during the entire study period, field notes, and documents obtained.

### Sample size calculations and analyses

As this study have 4 primary-end-points, i.e., the number of home deaths, use of specialized palliative care services, patient-reported quality of palliative care, and family-reported quality of palliative care, we have set alpha error of 0.0125 (two-sided) by the Bonferroni correction for multiple comparisons. All variables are compared between before and after the interventions using the Student t-test and Chi-square test, where appropriate.

#### Patient-reported quality of palliative care and family-reported quality of palliative care

To detect 0.2 effect size (one-fifth difference of standard deviation) for quality of palliative care (the Care Evaluation Scale) under statistical power of 0.8, 558 responses for each pre-intervention and post-intervention period is required for the analyses^35^. We have thus determined that 1500 patients and bereaved family members should be surveyed at each time in consideration of the estimated response rate (40-60%) and missing values (10%) [35].

#### The number of home deaths and use of specialized palliative care services

We first assumed 6% and 8% as each pre-intervention value from the national data, respectively, and we expected to achieve 12% and 20% as meaningful increases after the intervention, respectively; and this lead to the calculated sample size of 506 and 186 at the each point, respectively. In practice, as we survey all cancer death in 4 regions (5000), the sample size is sufficient.

## Discussion

This study is, to our knowledge, one of the most comprehensive evaluations of a region-based palliative care intervention program. This study has 3 major unique aspects.

First, as an outcome study, this study measures a wide range of outcomes, enabling comprehensive understanding and interpretation of the results. Especially, this study is outstanding in terms of: 1) we can obtain patient and family views as a nearly representative sample of advanced cancer patients and bereaved family members in the region; 2) we measure quality of care and quality of life specifically designed for palliative care populations, not heavily depending on the functional status; 3) we can know whether a patient actually died where they preferred, not only the location of death itself; and 4) we quantitatively measure the change in physicians' and nurses' knowledge, difficulties, and self-reported practice at regional levels, especially difficulties in communicating among local health care professionals. These efforts are all novel, and would provide useful insights to disseminate palliative care at regional levels as well as important information for designing future intervention studies in this area.

Second, this study adopts a qualitative process method. Rich description in each local context, understanding how the program works in shaping community palliative care through the grounded theory approach, and systematic collection of barriers and resolution will lead to obtaining deep insights into how quality community palliative care is developed. We will use the findings in two ways, namely as a practical guidance for clinicians and as an integrated information resource for policy makers.

Third, the intervention applied in this study is without a fundamental change in health care systems. This means the findings from this study would be applicable to all regions in Japan and probably in other countries, and clarify what is necessary for community palliative care as a basis. In other words, after completing this study, we can plan a randomized controlled trial using "novel" intervention requiring fundamental changes in the health care system.

The study analyses will be completed by the end of 2013, and a comprehensive understanding of the findings of this study will contribute to a deeper insight into how to develop community palliative care.

## Competing interests

The authors declare that they have no competing interests.

## Authors' contributions

TMo led the drafting of this paper and development of the protocol. MMi and TY co-designed the protocol from the view point of statistical specialists. AY and TMo co-designed qualitative process studies. NA, YK, MA, KH, MMa, MY, and YS co-designed the intervention protocol as specialists and/or local leaders. KE organized the study structure, funded all aspects of this study, and supervised all phases of this study. All authors read and approved the final draft.

## Pre-publication history

The pre-publication history for this paper can be accessed here:

http://www.biomedcentral.com/1472-684X/11/2/prepub
